# A Glove-Wearing Detection Algorithm Based on Improved YOLOv8

**DOI:** 10.3390/s23249906

**Published:** 2023-12-18

**Authors:** Shichu Li, Huiping Huang, Xiangyin Meng, Mushuai Wang, Yang Li, Lei Xie

**Affiliations:** Jiuli Campus, School of Mechanical Engineering, Southwest Jiaotong University, Chengdu 610031, China; lishichu2000@my.swjtu.edu.cn (S.L.); hphuang@swjtu.edu.cn (H.H.); 1151612378@my.swjtu.edu.cn (M.W.); yangl@swjtu.edu.cn (Y.L.); xielei@my.swjtu.edu.cn (L.X.)

**Keywords:** glove-wearing detection, YOLOv8, feature pyramid network, feature layer

## Abstract

Wearing gloves during machinery operation in workshops is essential for preventing accidental injuries, such as mechanical damage and burns. Ensuring that workers are wearing gloves is a key strategy for accident prevention. Consequently, this study proposes a glove detection algorithm called YOLOv8-AFPN-M-C2f based on YOLOv8, offering swifter detection speeds, lower computational demands, and enhanced accuracy for workshop scenarios. This research innovates by substituting the head of YOLOv8 with the AFPN-M-C2f network, amplifying the pathways for feature vector propagation, and mitigating semantic discrepancies between non-adjacent feature layers. Additionally, the introduction of a superficial feature layer enriches surface feature information, augmenting the model’s sensitivity to smaller objects. To assess the performance of the YOLOv8-AFPN-M-C2f model, this study conducted multiple experiments using a factory glove detection dataset compiled for this study. The results indicate that the enhanced YOLOv8 model surpasses other network models. Compared to the baseline YOLOv8 model, the refined version shows a 2.6% increase in mAP@50%, a 63.8% rise in FPS, and a 13% reduction in the number of parameters. This research contributes an effective solution for the detection of glove adherence.

## 1. Introduction

The manufacturing sector is among the industries with a high risk coefficient. The absence or reluctance to use appropriate safety gear (such as gloves) leaves workers unprotected in harsh working environments, posing safety risks and jeopardizing their physical well-being.

Reasons for workers not using gloves include the following: First, an inadequate awareness of how safety hazards can cause indirect injuries. For instance, in the Niger Delta, cement industry workers often operate in electrified environments without the protection of insulating gloves [1]. Second, workers lack relevant skill training; for example, wood factory workers in Calabar, southern Nigeria, believe personal protective equipment (PPE), such as gloves and safety boots, is beneficial, yet all respondents stated they lacked training on the proper use of PPE [2]. Third, the belief that gloves impede operations is common; a 46-year-old woman primarily relies on her fingertips to apply pressure and friction in her tasks after working for 18 years in a box factory. She feels gloves interfere with dexterous tasks, and thus rarely wears them. Consequently, she developed eczema and fissured dermatitis on her fingers [3].

Wearing gloves can effectively protect hands from environments prone to causing injury. Relying on manual checks for glove use undoubtedly wastes significant human resources. Hence, object detection algorithms present an optimal choice for detecting glove usage.

Current object detection algorithms can be broadly categorized into two main directions: two-stage detection and one-stage detection. Two-stage detectors include the likes of Faster R-CNN [4], R-FCN [5], and Mask R-CNN [6]. These algorithms generate a series of region proposals in images and subsequently classify and regress these proposals. Due to its bifurcated process, it is termed two-stage detection.

One-stage detectors primarily include YOLO [7,8,9,10], SSD [11], CornerNet [12], and M2Det [13], among others. Instead of generating proposal boxes, these algorithms directly predict object categories and locations in a single step. One-stage detection algorithms are typically faster than two-stage detectors due to their singular step execution, though they might compromise accuracy in some cases. Therefore, one-stage detection algorithms are better suited for tasks demanding high real-time performance and constrained computational resources.

With the rapid advancements in object detection algorithms, especially the immense success of the you only look once (YOLO) models in object detection, more researchers are venturing to apply object detection algorithms in real-world scenarios. For instance, Arunabha et al. [14] proposed an enhanced YOLOv5 model based on DenseNet and the Swin-Transformer detection head, achieving commendable results in road damage detection. Jiang S, Zhou X et al. [15] introduced the lightweight DWSC-YOLO model, incorporating DWS convolution and the Efficient attention mechanism, reducing the model size and making it apt for deployment on SAR radar devices. Sun C, Zhang S et al. [16] presented the MCA-YOLOV5-Light model for safety helmet detection, embedding the MCA module and implementing sparse training.

To avoid disturbing workers operating machinery, cameras are placed at a considerable distance from them. As a result, gloves occupy a small fraction of the image, with the shooting environment being intricate, leading to gloves being easily obscured by cluttered backgrounds. To address these challenges, this paper introduces the YOLOv8-AFPN-M-C2f model. The main contributions of this research are as follows:

1. This paper created a dataset for glove detection in factories. This dataset was collected in the production workshop of Zhengxi Hydraulic Company and consists of 2695 annotated high-resolution images depicting workers operating machinery with gloves or bare hands.
2. This study designed a new asymptotic feature pyramid network (AFPN) to replace the path aggregation feature pyramid network (PAFPN) network in YOLOv8. This facilitates the fusion of feature vectors between non-adjacent layers and reduces semantic contradictions between low-level and high-level features. Experiments show that the improved model detects glove targets more effectively.
3. This study added a superficial feature layer to the YOLOv8 model, which is rich in detailed image features, enhancing the model’s ability to perceive surface information and thereby improving the detection of glove image targets.

## 2. YOLOv8 Algorithm

**Figure 1:**
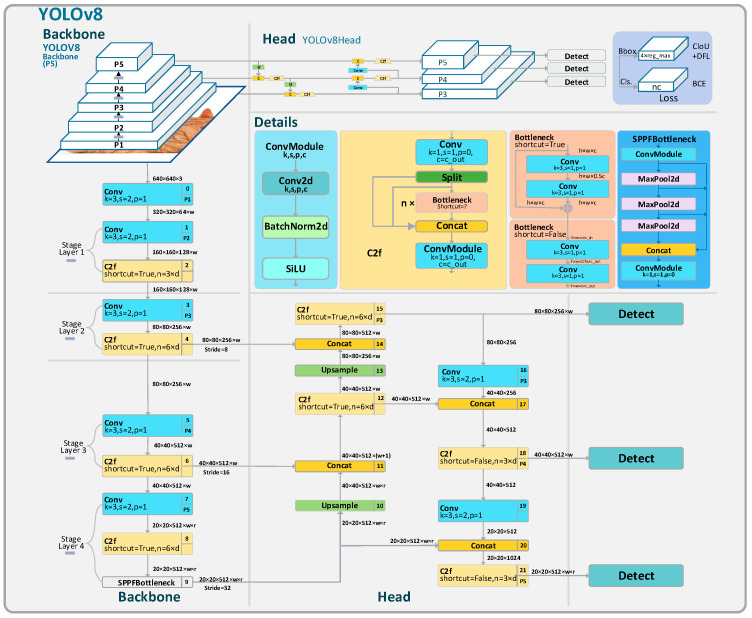
The structure of the YOLOv8 module.

The YOLOv8 algorithm is one of the more advanced object detection algorithms today. Its performance is so superior that it surpasses most other object detection algorithms. Therefore, this study has chosen YOLOv8 as the baseline of this study for comparison. As depicted in Figure 1, its backbone network employs the Darknet53 architecture, with the head utilizing PAFPN for feature fusion. The detection head adopts an anchor-free design. This anchor-free detection reduces the number of box predictions, thereby accelerating the speed of Non-Maximum Suppression (NMS), a complex post-processing step required to filter candidate detections after inference.

**Figure 2:**
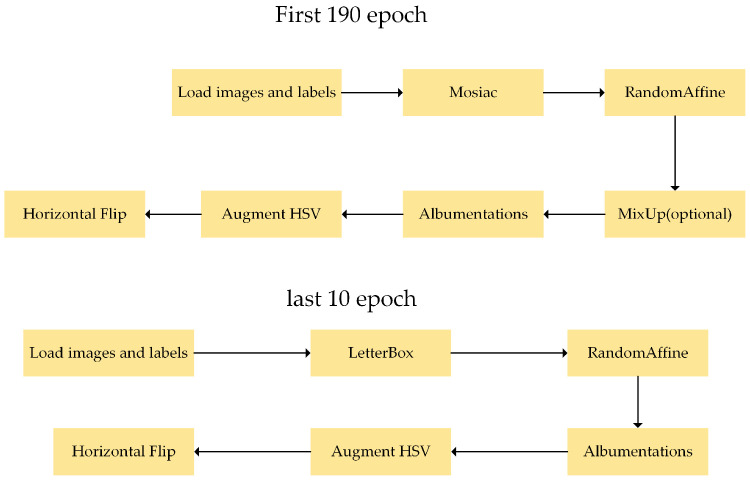
The model training procedure.

Regarding data augmentation, as shown in the model training workflow in Figure 2, YOLOv8 employs the Mosaic method, which enhances the dataset by randomly cropping and stitching images, thereby enhancing the model’s recognition capability. However, the Mosaic method may lead to overfitting issues. Therefore, in this study, Mosaic is turned off during the last 10 epochs of model training, allowing the model to complete its final convergence on a dataset of uncropped images to mitigate the potential drawbacks of the Mosaic data augmentation method. In terms of loss computation, recognizing the exceptional nature of the dynamic allocation strategy, YOLOv8 directly employs the TaskAlignedAssigner of task-aligned one-stage object detection (TOOD) [17]. The expression for TOOD is as follows:

(1) $$t=s^{\alpha }\times u^{\beta }$$

where s represents the predicted score corresponding to the annotated category, and u signifies the Intersection over Union (IoU) between the predicted and ground-truth boxes.

(2) $$loss\left(o,t\right)=-\frac{1}{n}(\underset{i}{\sum }\left(t\left[i\right]*\log\left(o\left[i\right]\right)+\left(1-t\left[i\right]\right)*\log\left(1-o\left[i\right]\right))\right)$$

where i denotes the sample label, *o*[*i*] is the model’s predicted probability for the sample, *t*[*i*] represents the actual probability of the sample, and *n* stands for the total number of samples.

YOLOv8 is the latest model in the YOLO series. Compared to the widely popular YOLOv5, YOLOv8 transitioned its first convolutional layer’s kernel from 6 × 6 to 3 × 3, and replaced the C3 module with the C2f module. The C2f module has more skip connections and additional split operations than the C3 module. The neck module has been streamlined by removing two convolutional layers. The most significant change is in the head section, transitioning from the original coupled head to a decoupled one, and the detection box has shifted from YOLOv5’s anchor-based to anchor-free.

## 3. Improved Algorithm: YOLOv8-AFPN-M-C2f

**Figure 3:**
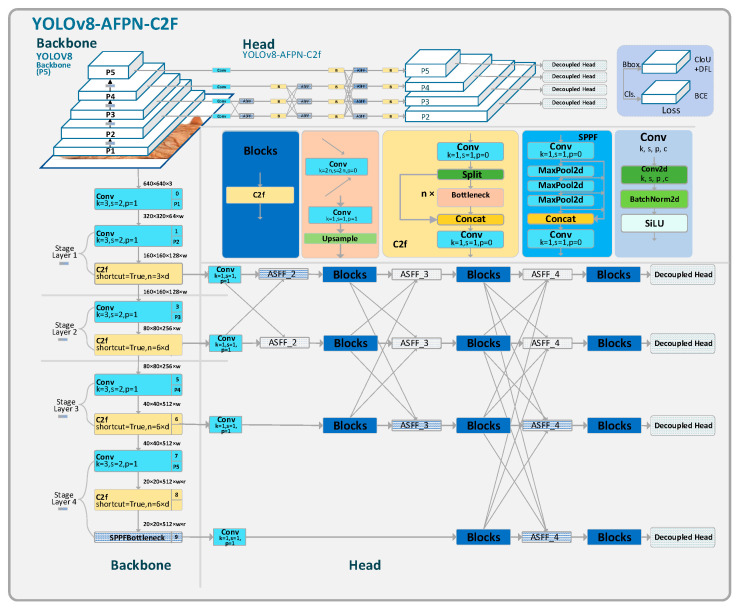
The structure of YOLOv8-AFPN-M-C2f.

YOLOv8-Asymptotic Feature Pyramid Network-More detection head-C2f modules (YOLOv8-AFPN-M-C2f) primarily made enhancements in the head and detection aspects. In the head section, This paper draws on the concept of AFPN [18] and proposes a new FPN design by replacing the conventional convolution blocks in the original architecture with C2f modules. In the backbone network, an additional feature layer is introduced, accompanied by an expanded detection head. Figure 3 illustrates the architectural design of the YOLOv8-AFPN-M-C2f model.

Compared to YOLOv8, the algorithm of this study possesses a more robust feature perception capability. It retains more superficial features and adds channels for feature information propagation, thereby improving accuracy. Additionally, it reduces the number of parameters, elevates frames per second (FPS), and decreases the demand for computational resources.

### 3.1. Feature Pyramid Network

Feature pyramid network (FPN) [19] was designed to tackle the challenge of multi-scale targets in object detection. At the heart of FPN lies the idea of constructing a hierarchical feature pyramid within Convolutional Neural Networks (CNNs), facilitating target detection across varying scales. FPN markedly enhances performance in tasks like object detection, keypoint detection, and semantic segmentation. It has been widely integrated into a myriad of networks such as RetinaNet [20], Mask R-CNN [6], Cascade R-CNN [21], EfficientDet [22], and Panet [23].

High-level features can be used to extract extensive characteristics from an image. However, they tend to overlook intricate details, thereby diminishing the model’s sensitivity to smaller targets. This often leads to suboptimal performance on datasets dominated by small objects. In contrast, low-level features focus on the rich, superficial details of an image, enabling the model to perceive localized nuances. Yet, these low-level features lack a holistic view. Within FPN, high-level features guide the intermediate ones, and the intermediate features, in turn, guide the low-level features. This cascading approach ensures the model is equipped with both a global perspective and localized focus, enhancing its predictive sensitivity. The FPN employs a bottom-up approach, transmitting high-level features to the lower layers, facilitating the fusion of features across different levels. However, during this transmission, high-level features remain uninfluenced by the low-level ones, posing a potential risk of information loss.

### 3.2. Improved FPN: AFPN-M-C2f

This study designs a progressive feature fusion pyramid network, named AFPN-M-C2f. This network can significantly reduce the number of parameters and enhance the feature information extraction ability. By minimizing ambiguities and conflicting information between features, it ultimately boosts the model’s prediction accuracy.

This network integrates features from each level with superficial features being fused with deeper ones in each iteration. Compared to the original AFPN, the AFPN-M-C2f adds an additional superficial feature layer and replaces the 3 × 3 convolution kernel in the Blocks feature extraction modules with C2f modules.

**Figure 4:**
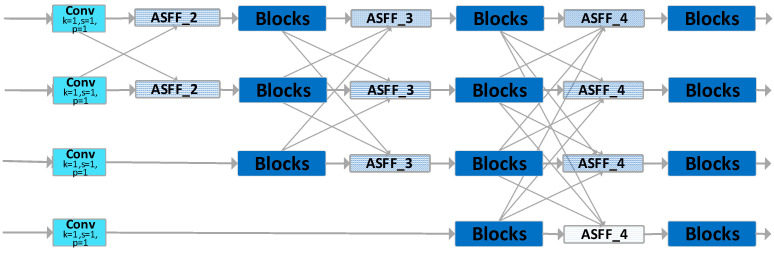
The structure of AFPN-M-C2f.

As depicted in Figure 4, AFPN extracts features layer by layer. Initially, during the primary stage, it integrates two feature vectors. In the intermediate phase, three feature vectors are merged, and in the final stage, four feature vectors are synergized, achieving a progressive fusion of features from low to high levels. Specifically, the network begins by integrating surface features, then delves into deeper features, and ultimately fuses abstract layer features. During this fusion process, arrows pointing diagonally upwards signify upsampling, while those pointing diagonally downwards indicate downsampling. The ASFF module adaptively fuses features from distinct layers, and the Blocks module is entrusted with feature extraction.

This paper employs AFPN-M-C2f to enhance the neck of YOLOv8, offering two notable advantages to the revamped YOLOv8:

1. It facilitates the fusion of features between non-adjacent layers, preventing the loss or degradation of features during their transmission and interaction.
2. It incorporates an adaptive spatial fusion operation, suppressing conflicting information between different feature layers and preserving only the useful features for fusion.

#### 3.2.1. Feature Vector Adjustment Module

**Figure 5:**
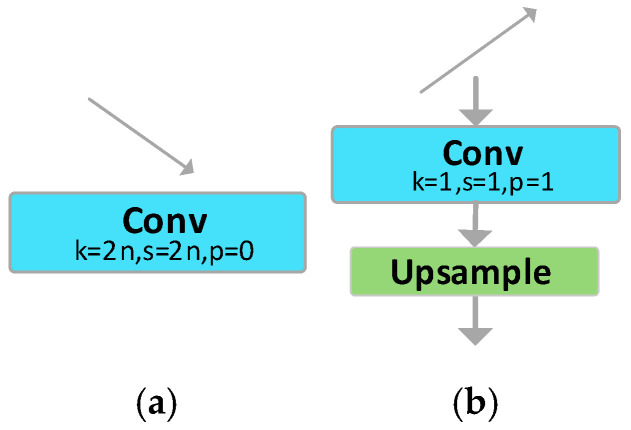
The Feature Vector Adjustment Module: (**a**) downsampling model; (**b**) upsampling model.

In the feature fusion process, feature vectors of different dimensions cannot be directly integrated; hence, it is imperative to adjust the dimensions of these feature vectors. AFPN employs 1 × 1 convolution and bilinear interpolation methods to upsample the features. As illustrated in Figure 5, a convolutional kernel of size n × n with a stride of n is used for downsampling. The size of n depends on the downsampling rate. For instance, a 2 × 2 convolution with a stride of 2 is used for 2× downsampling, a 4 × 4 convolution with a stride of 4 is utilized for 4× downsampling, and an 8 × 8 convolution with a stride of 8 is adopted for 8× downsampling.

#### 3.2.2. Adaptively Spatial Feature Fusion

**Figure 6:**
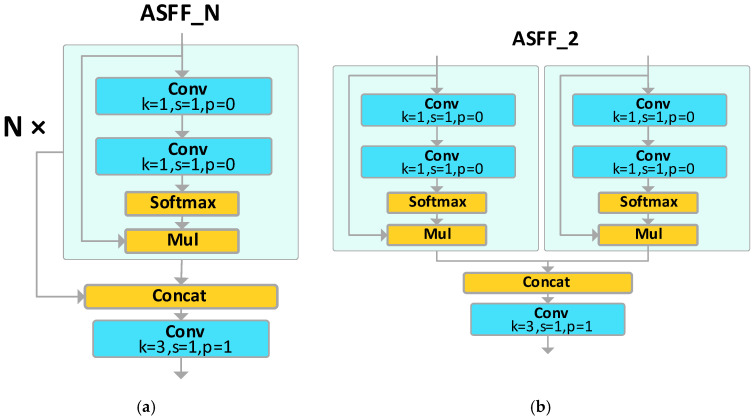
ASFF model: (**a**) ASFF_n; (**b**) ASFF_2.

In AFPN, a singular feature needs to integrate multiple features from other layers. To seamlessly integrate multi-level feature information, this paper draws inspiration from the Adaptive Spatial Feature Fusion Module (ASFF) [24], leading to the creation of the ASFF_N module. As shown as Figure 6a, N indicates the number of channels for feature fusion, where ASFF_N modules distribute the feature information of the N channels using a weighted approach. As depicted in Figure 6b, the ASFF_2′s features from the two input ends are weighted through two 1 × 1 convolutional kernels. These two weights are then combined, and, finally, a 3 × 3 convolutional kernel adjusts the size of the feature map to output the integrated feature.

To illustrate this with the ASFF_4 module as an example, the process of ASFF fusing four-channel features is represented as per Equation (3):

(3) $$f^{l}=w^{1\to l}\cdot x^{1\to l}+w^{2\to l}\cdot x^{2\to l}+w^{3\to l}\cdot x^{3\to l}+w^{4\to l}\cdot x^{4\to l}$$

where f denotes the feature vector fused by ASFF_4. The term $x^{n\to l}\left(n=1,\;2,\;3,\;4\right)$ refers to the feature vector on the feature transferred from the nth layer to the lth layer. The weights $w^{1\to l},w^{2\to l},w^{3\to l},w^{4\to l}$ represent the adaptively learned weights for the four distinct feature vectors directed to the lth layer.

The expression for the weights ($w^{1\to l},w^{2\to l},w^{3\to l},w^{4\to l}$) is shown as follows.

(4) $$w^{1\to l}+w^{2\to l}+w^{3\to l}+w^{4\to l}=1.$$

The normalization of the feature vectors is ensured by making the sum of weights for each vector equal to 1, thus preventing any unexpected amplification of or reduction in the vectors.

The ASFF module adeptly amalgamates features from multiple layers, diminishing semantic discrepancies and ambiguities between them, while retaining pertinent feature information.

#### 3.2.3. Enhancing the Feature Fusion Module of AFPN

In the realm of computer vision research, neural networks predominantly rely on convolutional kernels for feature extraction. These kernels are characterized by spatial invariance and channel specificity [25]. While spatial invariance ensures parameter efficiency across various spatial transformations, enlarging the kernel size leads to a substantial increase in parameter count. Stacking multiple kernels can circumvent this surge in parameters. However, such a practice also compromises computational efficiency.

**Figure 7:**
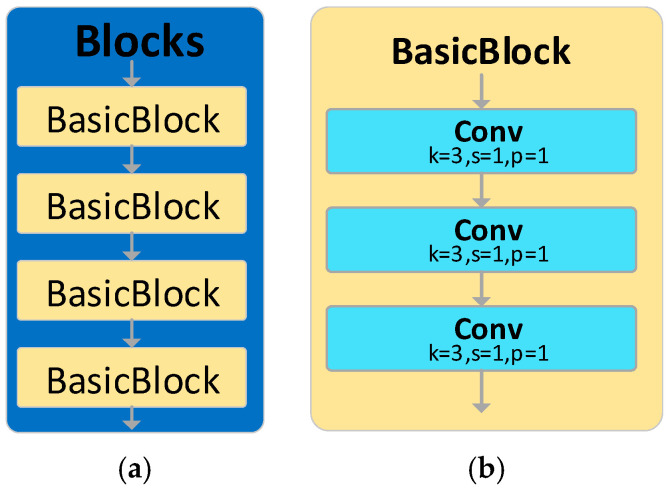
The original architecture of the feature extraction module in AFPN: (**a**) Blocks module; (**b**) BasicBlock module.

Within the AFPN framework, the Blocks module is employed for feature extraction. As depicted in Figure 7, the original Blocks is comprised of four BasicBlocks, each of which contains three 3 × 3 convolutional kernels, culminating in a total of 12 kernels for a single Blocks module. The abundance of kernels in the Blocks module results in an immense parameter count, consequently diminishing the effectiveness of feature extraction.

**Figure 8:**
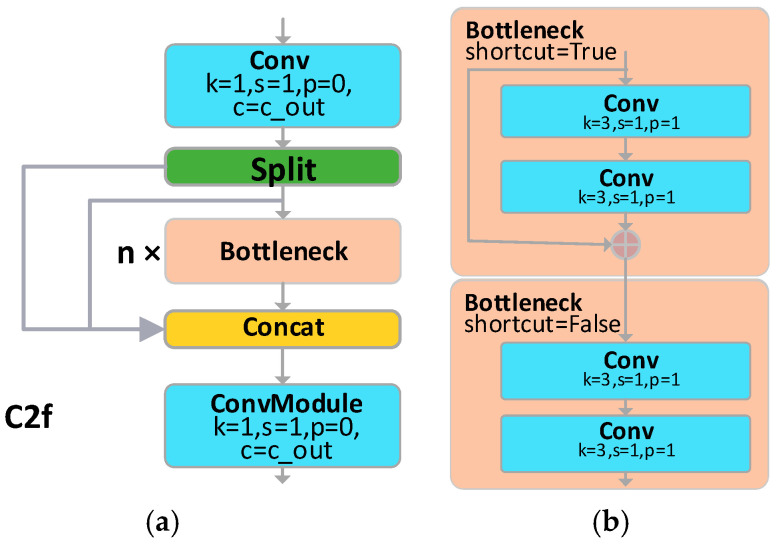
The architecture of the C2f module: (**a**) the structure of C2f; (**b**) Bottleneck in C2f.

In the YOLOv8-AFPN-M-C2f architecture, this study has incorporated a C2f module, supplanting the traditional Blocks module. This C2f module, distinct to YOLOv8, is pivotal in extracting features, thereby enhancing the efficacy of object detection. As delineated in Figure 8, the Bottleneck’s 3 × 3 convolution kernel within the C2f is entrusted with the task of harvesting feature data. The input feature information, traversing through a chain of sequentially linked Bottlenecks, transitions progressively from rudimentary feature maps to their advanced counterparts. While the elementary feature maps are replete with intricate details, they are devoid of overarching context. In contrast, the advanced feature maps imbue rich contextual cues but might sacrifice some minutiae. By establishing residual linkages between these diverse feature levels, the C2f module adeptly harnesses both the granular details and the encompassing context across various scales, thus amplifying the accuracy and robustness of object detection. Consequently, this paper employs the C2f module as a substitute for the Blocks module in AFPN.

### 3.3. More Feature Layers

**Figure 9:**
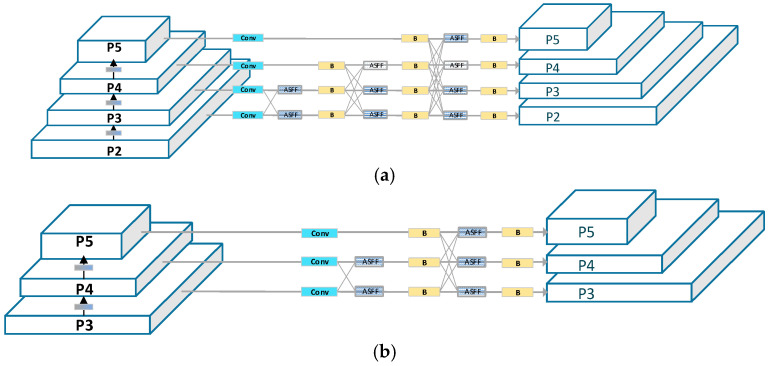
The feature fusion network architecture: (**a**) AFPN-M extracts features from the {P2, P3, P4, P5} layers of the main network. (**b**) Traditional FPN, exemplified by AFPN, extracts features from the {P3, P4, P5} layers.

FPN employs multi-scale feature maps to capture feature information across different resolutions. The conventional FPN extracts only the {P3, P4, P5} feature layers, with the advantage of having fewer parameters. Its shortcoming, however, is the limited perception of small objects, rendering it inadequate for detecting small items such as gloves; furthermore, it lacks sufficient semantic information, making it challenging to capture the semantic nuances in complex backgrounds like factory workshops. Taking YOLOv8-AFPN as an example, Figure 9b illustrates that the original AFPN only extracts the {P3, P4, P5} feature layers from the YOLOv8 backbone network.

This study introduces the AFPN-M network. As depicted in Figure 9a, the AFPN-M network extracts feature information from the {P2, P3, P4, P5} feature layers of the backbone network. Given the inclusion of additional feature layers, the network is aptly named AFPN-M.

Compared to the original AFPN network, the advantages of AFPN-M are manifold:

- The inclusion of the P2 feature layer, enriched with shallow feature information, enhances the model’s perceptibility of smaller objects and facilitates the transmission of surface feature vectors.
- An additional 16 feature layer transmission channels deeply integrate feature information.
- The introduction of five more Blocks modules allows for multi-dimensional, in-depth feature extraction and fusion.

## 4. Deep Learning Object Detection Datasets

**Figure 10:**
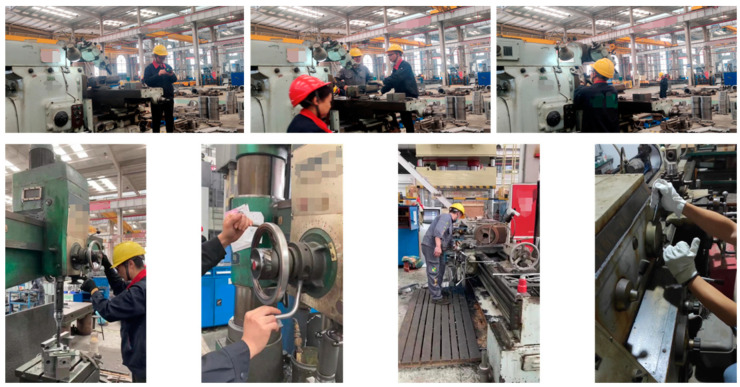
Unprocessed images from the Glove dataset.

The research utilizes the Glove dataset, gathered from the manufacturing workshop of the Zexi Hydraulic Company. This study annotated 2695 images that depict workers either wearing gloves or working barehanded during tasks such as equipment calibration and part machining on lathes, milling machines, and drilling machines (as shown in Figure 10). Within the Glove dataset, each instance is delineated by a rectangle and belongs to one of the five glove categories: Bare Hand, White Glove, Canvas Glove, or Black Glove. This dataset boasts the following advantages:

- The content collected closely mirrors the authentic working conditions of the workers, as researchers ventured directly into the Zexi Hydraulic Company’s workshop to capture the tasks performed by the staff.
- Compared to other similar datasets, the images of the study boast a much larger quantity, featuring several thousand images rather than merely a few hundred.
- The images are of pristine clarity with a high resolution of 3840 × 2160 pixels.

## 5. Training Methodology and Evaluation Metrics

### 5.1. Experimentation and Parameter Configuration

The experimental settings can be found in Table 1. For model training, researchers employed an AMD EPYC 7T83 64-Core Processor CPU and an RTX4090 GPU. The software environment includes CUDA version 11.8, Python 3.8, and Pytorch version 2.0.0.

In terms of hyperparameters, as depicted in Table 2, the researchers opted for the gradient-based SGD optimizer for model optimization. Concurrently, the researchers set an initial learning rate of 0.012, which dwindled to 0.0001 towards the end of the training. Additionally, to ensure stability and convergence speed during model training, the researchers established a momentum of 0.937 and a weight decay of 0.0005. The selection of these hyperparameters stems from multiple experimental results and precedents in research, ensuring the model’s commendable performance under varying conditions.

In the model training, specific parameters and hyperparameters were adopted to ensure optimal performance. As shown in Table 3, the researchers opted for an image size of 640 × 640 for training, with the number of iterations set at 200. Given computational efficiency and model convergence rate, the batch size was fixed at 64. 

To enhance the recognition ability of the model, the researchers adopted YOLOv8’s Mosaic method, which randomly crops four images and stitches them together as training data. The benefit of this approach is that it enriches the background of the images, and the model can train on four images at once, improving training efficiency and the model’s ability to learn background information. However, Mosaic may introduce some inaccurate annotations and lead to model overfitting issues. Therefore, the authors turned off Mosaic during the last 10 epochs of model training, enabling the training model to quickly complete label regression training on an uncropped image dataset. This approach is aimed at reducing the problems of inaccurate annotations and overfitting, thereby mitigating the potential downsides of data augmentation.

### 5.2. Evaluation Metrics

The authors employ evaluation metrics such as precision (P), recall (R), mean Average Precision (mAP), and frame per second (FPS) to comprehensively assess the model’s performance on the Glove dataset. Precision and recall are computed using the following formulas:

(5) $$P=\frac{TP}{TP+FP}$$

(6) $$R=\frac{TP}{TP+FN}$$

where P denotes the precision of the model’s predictions, R signifies the recall of the model’s predictions, TP represents the number of samples correctly classified as positive, FP indicates the number of samples incorrectly classified as positive, and FN represents the number of samples incorrectly classified as negative.

(7) $$AP=\int_{0}^{1}P\left(R\right)$$

(8) $$mAP=\frac{1}{C}\overset{C}{\underset{i=1}{\sum }}AP_{i}$$

(9) $$mAP@50\%=\frac{1}{C}\overset{C}{\underset{i=1}{\sum }}AP@0.5_{i}$$

where AP denotes the area under the precision–recall curve for a specific category at different confidence thresholds. mAP stands for the mean average precision, calculated by taking the average of the AP for each category. mAP@50% refers to the mAP with an intersection over union threshold of 0.5.

(10) $$FPS=\frac{1000}{time}$$

where FPS indicates the number of images the model processes per second, and time refers to the duration required for the model to process a single image, measured in milliseconds.

## 6. Results and Analysis of the YOLOv8-AFPN-M-C2f Algorithm

### 6.1. Comparative Analysis of Algorithmic Prediction Outcomes

**Figure 11:**
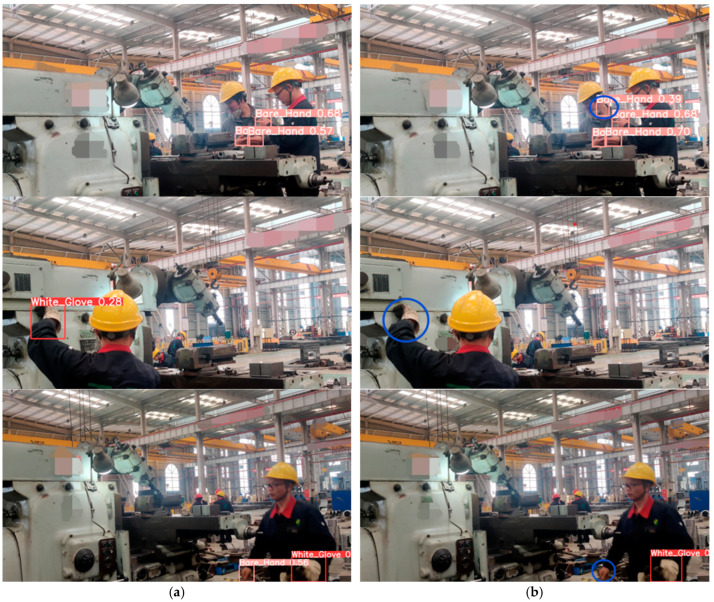
Predicted outcomes: (**a**) The images in the first column represent the prediction results of YOLOv8-AFPN-M-C2f. (**b**) The images in the second column depict the outcomes from YOLOv8 (baseline).

To vividly illustrate the enhancements of the modified YOLOv8 algorithm, this paper showcases the glove recognition results of YOLOv8-AFPN-M-C2f in comparison with the original YOLOv8. As observed from Figure 11, the YOLOv8 algorithm exhibited instances of false negatives (FN) and false positives (FP), which the authors have highlighted with blue circles in the figure. For instance, YOLOv8 mistakenly identified a worker’s neck as ‘Bare hand’ and overlooked certain gloves. However, these issues were adeptly addressed by the YOLOv8-AFPN-M-C2f algorithm.

### 6.2. Comparison Experiment

**Figure 12:**
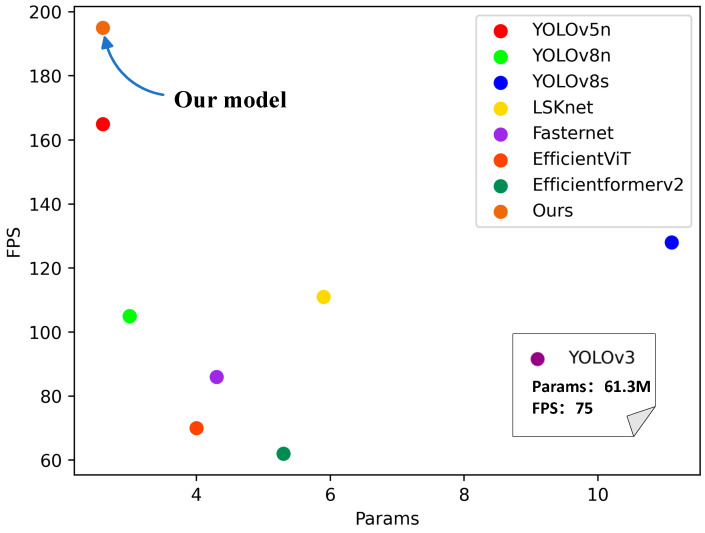
Comparison of FPS and Params performance across different models.

To validate the superiority of the algorithm proposed in this study on the Glove dataset, the researchers juxtaposed YOLOv8-AFPN-M-C2f with prevalent object detection algorithms, including YOLOv3, YOLOv5, YOLOv8n, YOLOv8s, LSKnet, Fasternet, EfficientViT, and Efficientformerv2. As depicted in Figure 12, the performance of YOLOv8-AFPN-M-C2f stands out impressively. Due to the large parameter size of the YOLOv3 model, the authors list its parameters and FPS separately, placing them in the bottom-right corner of the axis.

For a fair comparison of the inference performance of the models in Table 4 on the Glove dataset, the researchers replaced the YOLOv8 backbone network with Fasternet, EfficientViT, and Efficientformerv2, retaining YOLOv8’s head and detection modules. The experimental results, as illustrated in Table 4, ‘Performance evaluation of different algorithms on the Glove dataset’, show that compared to other models, the YOLOv8-AFPN-M-C2f model achieves the best performance in terms of mAP50%, FPS, and parameter quantity. Relative to the baseline model YOLOv8, the YOLOv8-AFPN-M-C2f model sees a 2.6% rise in mAP50%, a 63.8% surge in FPS, and a 13% decrease in model parameters. When juxtaposed with the YOLOv8s, which ranks second in mAP@50%, the YOLOv8-AFPN-M-C2f model has 77% fewer parameters. Compared to the similarly parameterized YOLOv5 model, the enhanced YOLOv8 model registers a 1.7% boost in mAP@50% and an 18% ascent in FPS. This underscores that the refined YOLOv8 boasts higher precision, superior real-time monitoring capability, and reduced hardware demands. Through these comparative metrics, the improvement in the YOLOv8 algorithm proposed in this study manifests in superior comprehensive performance, making it more fitting for deployment in resource-constrained factories for precise real-time glove-wearing detection.

### 6.3. Ablation Study

To validate the effectiveness of the various improvement modules in the enhanced YOLOv8 model, the researchers conducted several rounds of ablation studies. As shown in Table 5, the YOLOv8-AFPN-M-C2f model demonstrated the best performance.

In the experiments, the researchers initially selected the baseline model YOLOv8, which had an mAP50 of 0.952 and a parameter count of 3.01 M. Upon integrating the AFPN module into this model, its mAP50 increased to 0.964, with a slight reduction in parameter count to 2.74 M. This suggests that the AFPN can effectively enhance the model’s detection accuracy while optimizing its parameter count. Subsequently, the researchers tested the YOLOv8 + AFPN + C2f configuration, achieving an mAP50 of 0.942 and a parameter count of 2.31 M. Although the parameter count was marginally lower, there was a significant decrease in mAP50. This might indicate that the C2f module can efficiently reduce the model’s parameters, potentially at the cost of some detection accuracy. Ultimately, the YOLOv8-AFPN-M-C2f model, denoted as ‘Ours’, exhibited the best performance in all tests, achieving an mAP50 of 0.976 with a parameter count of 2.60 M. These findings demonstrate that the refined strategy achieves an optimal balance when considering both detection accuracy and model complexity.

### 6.4. The Experiments of Methods to Enhance AFPN

#### 6.4.1. Experiments with Various Feature Extraction Modules Replacing Blocks

In this study, the authors enhanced the AFPN network by replacing the Blocks module with the C2f module. To illustrate the superiority of the C2f module over other feature extraction modules, the authors conducted comparative experiments in which various network feature extraction modules substituted the Blocks in AFPN. Specifically, researchers replaced the original Blocks in AFPN with the CloAtt, Faster, VoVGSCSP, DBB, and C3 modules, respectively.

**Figure 13:**
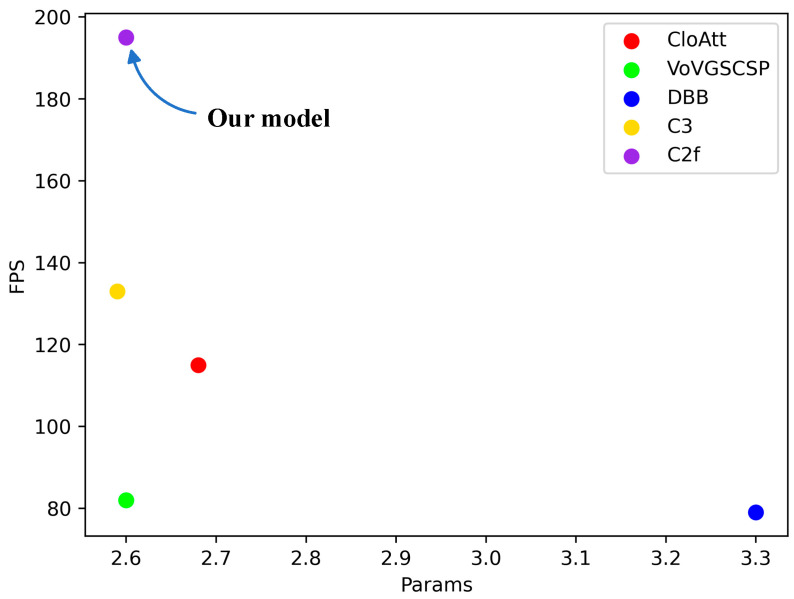
Scatter plot of parameters-FPS for different modules replacing blocks.

As presented in Table 6, the model employing C2f in place of Blocks led the pack in both mAP50% and FPS. It achieved a 1% higher mAP50% compared to the best performing VoVGSCSP module and outperformed the most lightweight C3 module by 48% in terms of FPS. Figure 13 illustrates the relationship between the parameter count and FPS for each model. The graph underscores that the AFPN network equipped with C2f exhibits optimal performance, underscoring that C2f is indeed the most suitable feature extraction module to replace Blocks in AFPN.

#### 6.4.2. Number of C2f Modules in Series

This section examines the impact on model performance when varying the number of C2f modules in series to replace the Blocks. As seen in Table 7, under all test conditions such as Bare hand, White glove, Canvas glove, and Black glove, the model with a single C2f cascade always exhibits higher mAP50 performance. Specifically, when the number of C2f cascades is 1, the overall mAP50 of the model is higher than the other configurations. With the increase in the number of C2f cascades, there is a declining trend in mAP50 performance, especially evident in configurations with three and four C2fs.

From the FPS and Params data, it is evident that as the number of C2f cascades increases, FPS gradually declines, and the model’s parameter count ascends. This suggests that an escalation in model complexity, while adding computational and storage overheads, does not yield a commensurate boost in performance.

In summary, compared to the more intricate multi-cascade C2f structures, the single C2f cascade model consistently exhibits superior performance across all test conditions.

### 6.5. Futher Dicussion

The above experimental analysis results confirm the effectiveness of the proposed method in detecting whether workers are wearing gloves. More specifically, compared to existing methods, the proposed method has been proven to be the most advanced in glove image object detection, providing a solid research foundation for glove object detection in practical engineering applications. However, there are still some issues with the proposed method that can be improved and discussed in future research, summarized as follows:

Although the analysis results indicate that the proposed method can enhance the accuracy and smoothness of identifying whether workers are wearing gloves, in practical applications, the shooting angles of image sensors vary (e.g., top-down and bottom-up views). Therefore, in future research, the proposed method can be applied to analyze glove detection using image sensors under top-down and bottom-up working conditions. Additionally, the computational efficiency of the proposed method for detecting glove-wearing targets when the number of image sensors increases is unknown. Hence, using the proposed method to study glove image detection under various working conditions as the number of image sensors increases is worthy of attention in future work. The authors anticipate employing transfer learning to enable the model to precisely detect glove wearing in both top-down and bottom-up working conditions. The authors also plan to enhance the model’s capability to recognize data from multiple image sensors by streamlining the network parameters of the model.

Currently, some new object detection methods (such as attention mechanisms and sparse autoencoders) have been widely applied in the field of image detection. However, their efficacy in enhancing the performance of glove object detection is not yet known. Therefore, future research work can focus on combining data-driven methods with the proposed YOLOv8-AFPN-M-C2f to achieve the goal of improving the efficiency of glove object detection.

## 7. Conclusions

Glove detection in workshops is confronted with the challenges of limited computational resources on edge devices and intricate backgrounds. To tackle these issues, this study introduces the YOLOv8-AFPN-M-C2f model. This model preserves the YOLOv8’s backbone network and substitutes its head with AFPN. An added feature layer, enriched with shallow feature information, and the employment of the C2f module boost AFPN’s feature extraction prowess. Moreover, the authors delved into the experimental exploration of the number of concatenated C2f modules. Ultimately, the researchers validated the enhanced YOLOv8’s performance on a glove dataset and, through comparative experiments with contemporary advanced models, determined that the YOLOv8-AFPN-M-C2f model achieved exemplary outcomes in mAP@50%, parameter count, and FPS.

While the YOLOv8-AFPN-M-C2f model has reduced the parameter size and enhanced FPS, deploying it to edge devices for smooth and stable object detection in scenarios with extremely scarce computational resources remains a challenging endeavor. The researchers will investigate methods to significantly reduce the model’s parameter size without compromising its accuracy, ensuring its adaptability to environments with critically limited computing resources.

## Figures and Tables

**Table 1:** Experimental environment configuration.

Experimental Component	Version
OS	Ubuntu20.04
CPU	AMD EPYC 7T83 64-Core Processor
GPU	RTX4090
CUDA version	11.8
Python version	3.8
Pytorch version	2.0.0

**Table 2:** The hyperparameters for training.

Hyperparameter	Value
gradient-based optimizers	SGD
initial learning rate (lr0)	0.012
final learning rate	0.0001
momentum	0.937
weight decay	0.0005

**Table 3:** Parameters for model training.

Parameter Name	Setting
Image dimensions	640 × 640
Number of epochs	200
Batch size	64
Data augmentation method	Mosaic

**Table 4:** Performance evaluation of different algorithms on the Glove dataset.

Model	mAP50/(%)							FPS	Params/M
	Bare Hand	White Glove	Canvas Glove	Black Glove	Glove Avg ^1^	P	R		
YOLOv3 [9]	0.945	0.944	0.976	0.987	0.969	0.974	0.915	77	61.3
YOLOv5 [26]	0.935	0.929	0.971	0.990	0.963	0.967	0.917	165	2.6
YOLOv8 [27]	0.941	0.921	0.952	0.993	0.955	0.955	0.897	105	3.0
YOLOv8s [27]	0.957	0.953	0.984	0.981	0.973	0.952	0.932	128	11.1
LSKnet [28]	0.926	0.924	0.966	0.981	0.957	0.958	0.914	111	5.9
Fasternet [29]	0.948	0.959	0.945	0.974	0.960	0.961	0.909	86	4.3
EfficientViT [30]	0.914	0.926	0.979	0.982	0.962	0.956	0.936	70	4.0
Efficientformerv2 [31]	0.935	0.915	0.964	0.992	0.956	0.951	0.915	62	5.3
Ours	0.960	0.965	0.984	0.993	0.980	0.975	0.937	195	2.6

^1^ Gloves avg signifies the average mAP@50% of the model, which is the average mAP50% of the three categories: White glove, Canvas glove, and Black glove.

**Table 5:** Experimental results of the ablation experiment.

Model	AFPN	More Detect Head	C2f	mAP50 ^1^ (%)	Params/M
YOLOv8n(baseline)				0.952	3.01
YOLOv8+AFPN	√			0.964	2.74
YOLOv8+AFPN+ more Detect head	√	√		0.956	3.00
YOLOv8+AFPN+C2f	√		√	0.942	2.31
Ours	√	√	√	0.976	2.60

^1^ mAP50 refers to the comprehensive mAP@50% of the model, which is the average mAP50% for the four categories: Bare hand, White glove, Canvas glove, and Black glove.

**Table 6:** Evaluation of performance using different modules to replace blocks.

Model	mAP50/(%)							FPS	Params/M
	Bare Hand	White Glove	Canvas Glove	Black Glove	Glove Avg ^1^	P	R		
CloAtt [32]	0.931	0.957	0.958	0.995	0.970	0.963	0.921	115	2.68
VoV-GSCSP [33]	0.954	0.955	0.959	0.987	0.967	0.972	0.924	82	2.60
DBB [34]	0.934	0.948	0.943	0.995	0.952	0.962	0.909	79	3.30
C3 ^2^ [26]	0.939	0.889	0.940	0.995	0.941	0.965	0.895	133	2.59
C2f(ours)	0.960	0.965	0.984	0.993	0.980	0.975	0.937	195	2.60

^1^ Glove avg signifies the average mAP@50% of the model, which is the average mAP50% of the three categories: White glove, Canvas glove, and Black glove. ^2^ C3 is the module responsible for feature extraction in YOLOv5.

**Table 7:** The influence of the number of C2f modules in Blocks on the model’s performance.

The Number of C2f	mAP50/(%)							FPS	Params/M
	Bare Hand	White Glove	Canvas Glove	Black Glove	Glove Avg ^1^	P	R		
1(ours)	0.960	0.965	0.984	0.993	0.980	0.975	0.937	195	2.60
2	0.940	0.941	0.960	0.983	0.961	0.963	0.904	102	2.65
3	0.958	0.953	0.959	0.989	0.967	0.964	0.916	89	2.70
4	0.945	0.928	0.961	0.990	0.960	0.960	0.909	77	2.75

^1^ Glove avg refers to the comprehensive mAP@50% of the model, which is the average mAP50% for the three categories: White glove, Canvas glove, and Black glove.

## Data Availability

The foundational data for this article were derived from Chengdu Zhengxi Hydraulic Press company. The derived data generated in this study will be shared by the respective authors upon reasonable request.
